# Sulfur‐Embedded Pure Green Multiple Resonance TADF Emitters: Optimizing Photophysical and Electroluminescent Properties

**DOI:** 10.1002/advs.202512796

**Published:** 2025-10-29

**Authors:** Chao Jiang, Yufang Nie, Chi Cao, Xiaoxian Song, Jie Liang, Xuming Zhuang, Zhiqiang Li, Baoyan Liang, Yue Wang

**Affiliations:** ^1^ Department of Materials Science and Technology Jihua Laboratory 28 Huandao Nan Road Foshan Guangdong Province 528000 P. R. China; ^2^ State Key Laboratory of Supramolecular Structure and Materials College of Chemistry Jilin University 2699 Qianjin Street Changchun Jilin Province 130012 P. R. China

**Keywords:** benzothienocarbazole, efficiency roll‐off, multiple resonance thermally activated delayed fluorescence, organic light‐emitting diodes, pure green emission

## Abstract

Two pure green multiple resonance thermally activated delayed fluorescence (MR‐TADF) emitters are reported here by utilizing benzothienocarbazole groups with sulfur atoms connecting with the 3‐position carbon atoms of carbazole units, which are named Th‐Cz‐BN3 and Th‐Cz‐BN6. Although their molecular structures are similar, significant differences in energy splitting and spin‐orbit coupling between the singlet and triplet excited states are exhibited, resulting from the differences in steric configurations and consequent electronic structures. The Th‐Cz‐BN3 and Th‐Cz‐BN6‐based devices display pure green emission with Commission Internationale de L'Eclairage (CIE) y values exceeding 0.7 and maximum external quantum efficiencies of over 34.0%. Moreover, the Th‐Cz‐BN3‐based non‐sensitized device exhibits remarkable efficiency roll‐offs of 0.3 and 16.8% at 100 and 1000 cd m^−2^, respectively, which can be ascribed to the rapid rates of radiative transition and reverse intersystem crossing processes. The results not only achieve sulfur‐embedded pure green MR‐TADF emitters but also pave the way for designing highly efficient MR‐TADF emitters with suppressed efficiency roll‐offs.

## Graphical abstract

Sulfur atoms are inserted into the multiple‐resonance thermally activated delayed fluorescence framework for developing pure green emitters. The analogous isomers present distinguishing photophysical and electroluminescent properties. The better emitter‐based non‐sensitized device exhibits a maximum external quantum efficiency of 36.4% and a remarkable efficiency roll‐off of 16.8% at 1000 cd m^−2^.

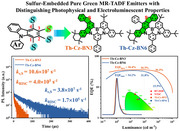

## 1 Introduction

Multiple resonance thermally activated delayed fluorescence (MR‐TADF) materials, proposed by Hatakeyama and co‐workers, exhibit narrowband emission and high efficiencies owing to their rigid conjugate skeleton and multiple resonance distribution of the frontier molecular orbitals, which are the potential substitutes for phosphor materials, especially in the Broadcast Television 2020 (B.T. 2020) color gamut for ultra‐high‐definition (UHD) displays.^[^ ^1^ ^]^ The intrinsic narrowband emission spectra of MR‐TADF emitters eliminate the need for optical filters and microcavity structures of the organic light‐emitting diode (OLED) devices, which decreases the energy loss in the spectra‐narrowing processes and increases the device efficiencies.^[^ ^2^ ^]^ Otherwise, the pure organic MR‐TADF materials possess the advantages of low cost and environmental sustainability compared with the current widely used precious metal‐based phosphor materials.^[^ ^3^ ^]^

**Scheme 1:**
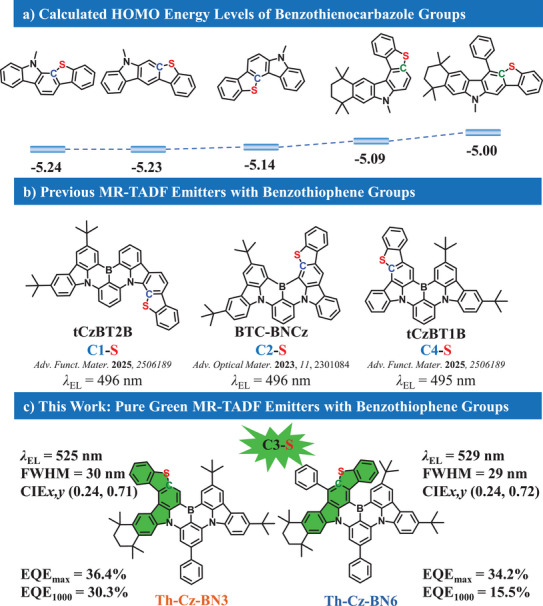
a) Calculated HOMO energy levels of benzothienocarbazole groups. b) Reported MR‐TADF emitters with benzothiophene groups. c) The chemical structures and EL properties of Th‐Cz‐BN3 and Th‐Cz‐BN6 in this work.

Although many MR‐TADF emitters have been reported and some of them demonstrated B.T. 2020 emission spectra, the efficiency roll‐offs of MR‐TADF emitter‐based devices remain a significant challenge.^[^ ^4^, ^5^, ^6^, ^7^, ^8^, ^9^, ^10^, ^11^, ^12^, ^13^, ^14^, ^15^ ^]^ The long‐lifetime triplet excitons and the slow rate of reverse intersystem crossing (*k*_RISC_) are responsible for the severe triplet‐triplet annihilation (TTA), singlet‐triplet annihilation (STA), and triplet‐polaron annihilation (TPA) at high current densities.^[^ ^16^ ^]^ Owing to the competition of the radiative transition of the singlet and the RISC process of the triplet, the *k*_RISC_ is hard to improve owing to the rigid conjugate framework‐induced rapid rate of radiative transition (*k*_r,S_). Great efforts have been made to improve the *k*_RISC_ of MR‐TADF emitters.^[^ ^17^, ^18^, ^19^ ^]^ On the one hand, extending the MR conjugate skeleton with building blocks effectively decreases the energy splitting between the first singlet and triplet (Δ*E*_ST_) and enhances *k*_RISC_.^[^ ^20^, ^21^, ^22^ ^]^ On the other hand, introducing heavy atoms (S, Se) into the framework of the MR‐TADF emitters is also effective in improving the spin‐orbit coupling (SOC) effect and increasing the *k*_RISC_.^[^ ^23^, ^24^, ^25^ ^]^ However, the different positions of the heavy atoms in the MR‐TADF frameworks contribute to significant differences in energy levels of the excited states, full width at half height (FWHM) of the emission spectra, and the SOC effect.^[^ ^26^, ^27^, ^28^, ^29^ ^]^ Sulfur atoms embedded into the MR‐TADF framework in benzothienocarbazole groups can achieve synergistic improvements of *k*_r,S_, and *k*_RISC_. However, in the reported benzothienocarbazole groups utilized in MR‐TADF emitters, the sulfur atoms were connected with carbon atoms on carbazole 1‐, 2‐, and 4‐positions.^[^ ^26^, ^27^ ^]^ The highest occupied molecular orbital (HOMO) energy levels of these benzothienocarbazole groups are deep, and the target MR‐TADFemitters demonstrated blue‐green emissions with electroluminescence peaks at ≈500 nm, as shown in **Scheme** **1**a,b, which are not fit for direct utilization for displays.

The theoretical calculation results of the benzothienocarbazole groups with different locations of the sulfur atoms have been performed, and the results are shown in Figure S1 (Supporting Information). ThCz‐4 and ThCz‐5 demonstrated shallower HOMO energy levels compared with other groups, indicating their stronger electron‐donating ability and potential for constructing pure‐green MR‐TADF emitters. Moreover, the triplet energy levels of ThCz‐4 and ThCz‐5 were also smaller than other groups, which would be fit for constructing local triplet excited states in green emitters, and hence larger SOC constants and more rapid *k*_RISC_. As a consequence, ThCz‐4 and ThCz‐5 were employed to construct pure green MR‐TADF emitters. As shown in Scheme 1c, Th‐Cz‐BN3 and Th‐Cz‐BN6 have been designed and synthesized, which present pure green emission with peaks at 517 and 523 nm in dilute solutions, along with FWHM of 24 and 26 nm for Th‐Cz‐BN3 and Th‐Cz‐BN6, respectively. Th‐Cz‐BN3 exhibits concentration‐dependent emission spectra owing to its more planar configuration and obvious aggregation effect, while Th‐Cz‐BN6 presents concentration‐independent emission spectra with an analogous helicene structure and steric‐hindrance effect. Ultimately, Th‐Cz‐BN3‐ and Th‐Cz‐BN6‐based non‐sensitized devices exhibit pure green emission with peaks at 525 and 529 nm, along with FWHM of 30 and 29 nm, CIE coordinates of (0.24, 0.71) and (0.24, 0.72), respectively. Moreover, the Th‐Cz‐BN3‐based non‐sensitized device presents much more suppressed efficiency roll‐off of 16.8% at 1000 cd m^−2^, which is 54.7% for the Th‐Cz‐BN6‐based one. The results demonstrate that the sulfur‐embedded positions in MR‐TADF frameworks significantly influence the energy levels, photophysical properties, and electroluminescent properties.

## 2 Results and Discussion

### 2.1 Synthesis, Representation, Thermal, and Electrochemical Properties

**Figure 1:**
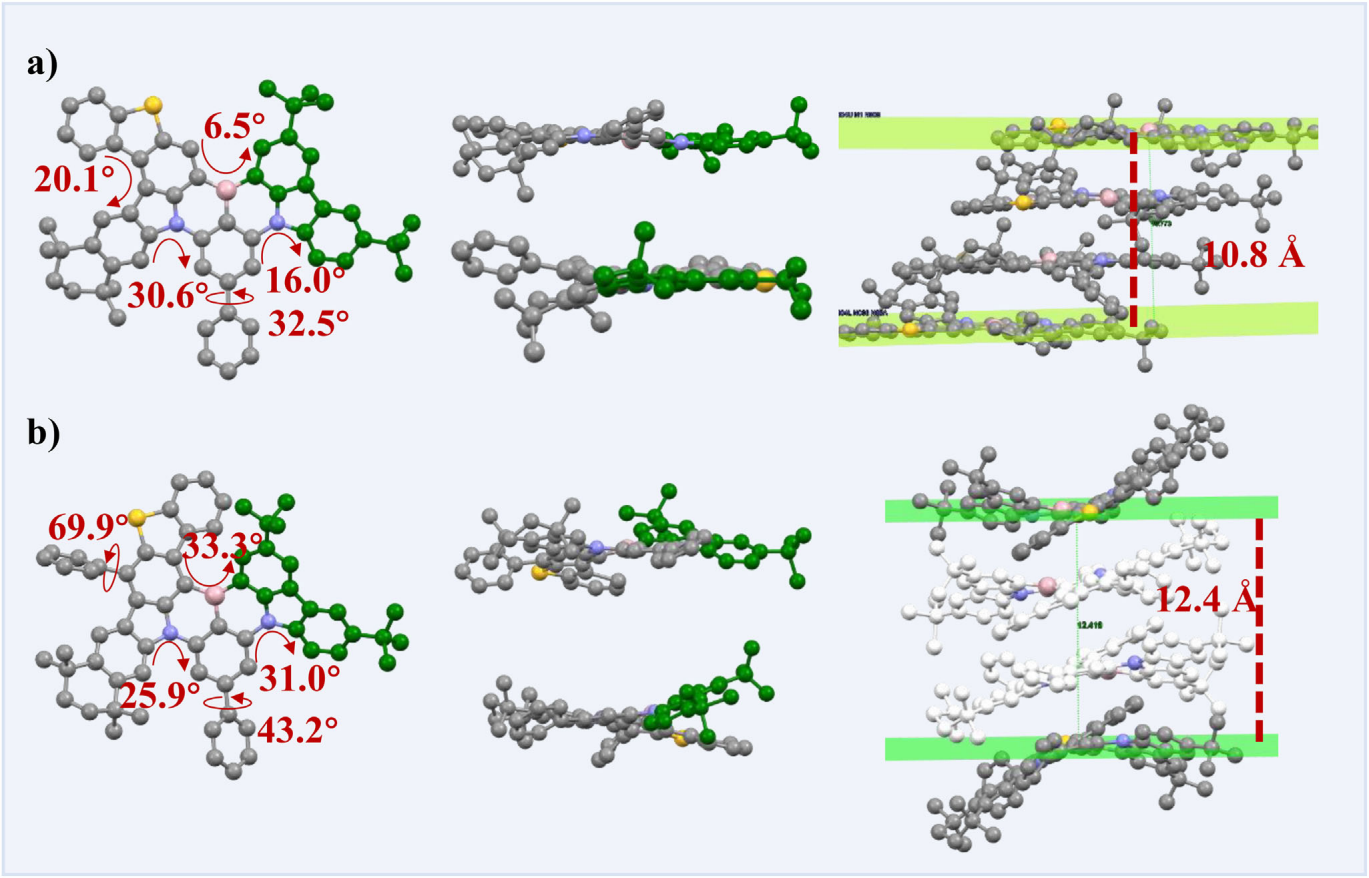
Top view, side view, and stacking structures of Th‐Cz‐BN3 a) and Th‐Cz‐BN6 b) in single crystals. CCDC no: 2452814 for Th‐Cz‐BN3 and 2449214 for Th‐Cz‐BN6.

The synthetic routes of Th‐Cz‐BN3 and Th‐Cz‐BN6 are illustrated in Scheme S1 (Supporting Information). The tetramethylcyclohexyl and hanging phenyl units in benzothienocarbazole groups were introduced to construct a steric hindrance and restrain the generation of isomerides. The target molecules have been characterized by ^1^H NMR, ^13^C NMR, and mass spectrometry (Figures S2–S21, Supporting Information). The single crystals of Th‐Cz‐BN3 and Th‐Cz‐BN6 were obtained by solvent diffusion from dichloromethane and methyl alcohol, which further verified the structures of Th‐Cz‐BN3 and Th‐Cz‐BN6, as shown in **Figure** **1**. Th‐Cz‐BN3 presented a more planar configuration than Th‐Cz‐BN6, with torsional angles less than 32.5 °. The distance of four stacked Th‐Cz‐BN3 was 10.8 Å, which was smaller than that of Th‐Cz‐BN6 (12.4 Å), demonstrating more severe *π–π* intermolecular interactions of Th‐Cz‐BN3. As for Th‐Cz‐BN6, the torsional angles of the segments were much larger. The torsional angle between the hanging phenyl units on benzothienocarbazole and benzothienocarbazole groups was ≈70 °, and the torsional angle of the analogous helicene structure was 33.3 °, exhibiting a more twisted configuration. A couple of helical chiral isomers of Th‐Cz‐BN6 were obtained in the single crystal (Figure S22, Supporting Information), which failed to be separated via chiral column chromatography. The thermal properties of the compounds were represented by thermogravimetric analysis (TGA) and differential scanning calorimeter (DSC), which demonstrated high thermal decomposition temperatures (*T*_d,5%_) of 497 and 511 °C for Th‐Cz‐BN3 and Th‐Cz‐BN6, respectively, along with glass transition temperatures (*T*_g_) of 235 and 212 °C (Figure S23, Supporting Information). The pretty good thermostability of the compounds suggests they are suitable for device fabrication by vacuum evaporation. Cyclic voltammetry (CV) measurements have been applied to test the electrochemical stability and determine the energy levels of the compounds (Figure S24, Supporting Information). Reversible CV curves of the compounds indicated their electrochemical stability, and the calculated energy levels of HOMO were −5.19 and −5.12 eV of Th‐Cz‐BN3 and Th‐Cz‐BN6, respectively, along with the lowest unoccupied molecular orbital (LUMO) energy levels of −2.88 and −2.94 eV. As a result, Th‐Cz‐BN6 showed a narrower energy gap than Th‐Cz‐BN3 and indicated a more red‐shifted emission spectrum of Th‐Cz‐BN6.

### 2.2 Theoretical Calculations

**Figure 2:**
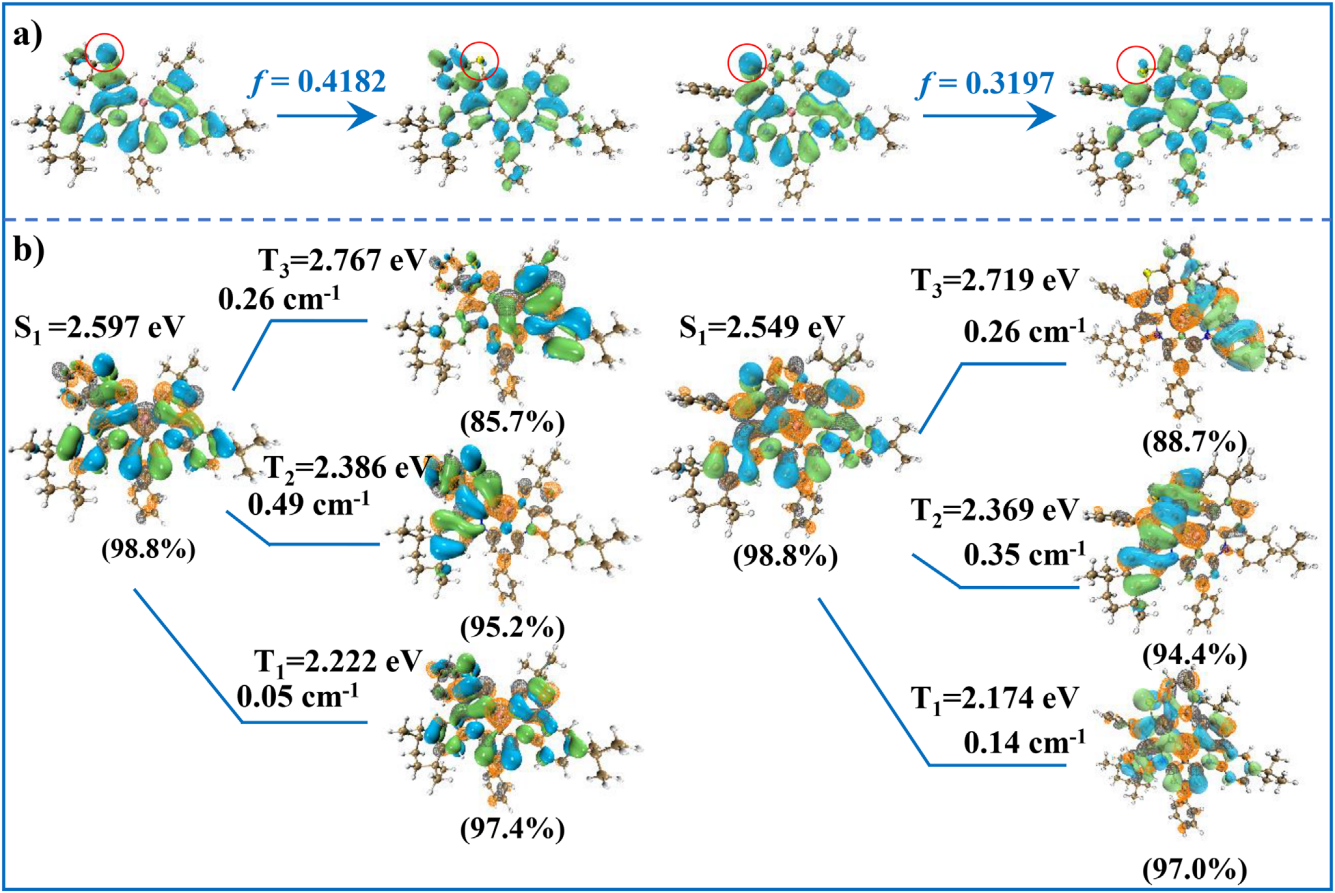
a) Distributions of the frontier orbitals and oscillator strength of Th‐Cz‐BN3 and Th‐Cz‐BN6. b) NTO analysis (blue and green for hole; orange and gray for particle), energy levels, and SOC constants of Th‐Cz‐BN3 and Th‐Cz‐BN6.

The optimized ground state (S_0_) configurations and distributions of the frontier orbitals of the compounds were calculated by density functional theory (DFT) performed at B3LYP/ 6‐31G (d, p) level, and the energy levels of the excited states were also calculated with the same methods. As illustrated in **Figure** **2**a, the frontier orbital distributions of Th‐Cz‐BN3 and Th‐Cz‐BN6 demonstrated a typical MR distribution with HOMO distributed on nitrogen atoms and carbon atoms at ortho‐/para‐positions and LUMO located at boron atom and their ortho‐/para‐carbon atoms. The sulfur atoms were mainly located at HOMO sites. The oscillator strength of Th‐Cz‐BN3 was 0.4182, and it was larger than that of Th‐Cz‐BN6 (0.3197), which could be ascribed to the more planar and rigid configuration, and it was beneficial for rapid k_r,S_. The natural transition orbitals (NTO) analysis results are shown in Figure 2b. The S_1_ and T_1_ excited states presented similar transformations with S_0_ from hole to particle, and the spin‐orbit coupling (SOC) constants were as small as 0.05 and 0.14 cm^−1^, for Th‐Cz‐BN3 and Th‐Cz‐BN6, respectively. However, the hole distributions of T_2_ excited states for Th‐Cz‐BN3 and Th‐Cz‐BN6 were localized at benzothienocarbazole groups, while the particle maintained MR distributions. The difference in NTO distributions between S_1_ and T_2_ contributed to larger SOC constants of 0.49 cm^−1^ for Th‐Cz‐BN3 and 0.35 cm^−1^ for Th‐Cz‐BN6. The larger SOC constant between S_1_ and T_2_ of Th‐Cz‐BN3 could be ascribed to the larger contributions of the sulphur atom in the T_2_ transition, as shown in Table S1 (Supporting Information). The larger SOC constant of Th‐Cz‐BN3 indicated a more rapid k_RISC_. Moreover, the T_3_ excited states of Th‐Cz‐BN3 and Th‐Cz‐BN6 demonstrated similar hole and particle distributions. The particle maintained MR distributions, while the hole was mainly located at the 3,6‐di‐tert‐butylcarbazole units. As a result, the same SOC constants of 0.26 cm^−1^ were obtained between S_1_ and T_3_ excited states of Th‐Cz‐BN3 and Th‐Cz‐BN6. Further analyses of hole and electron distributions were performed, and the results are presented in Figures S25–S27 (Supporting Information).^[^ ^30^ ^]^ Similar hole and electron distributions were observed in the T_1_ and S_1_ excited states, which represented typical MR distributions. As for T_2_ and T_3_ excited states, large overlaps of hole and electron were obtained on benzothienocarbazole and 3,6‐di‐tert‐butylcarbazole units, respectively, resulting in large SOC constants between T_2_/T_3_ and S_1_ excited states. A detailed hole‐electron analysis of the high‐level excited states of Th‐Cz‐BN3 and Th‐Cz‐BN6 was also performed, and the results are listed in Tables S2 and S3 (Supporting Information). Both of the emitters demonstrated the largest SOC constants between T_2_ and S_1_, as well as small energy splittings, indicating the RISC processes from T_2_ to S_1_. According to theoretical calculation results, Th‐Cz‐BN3 would exhibit both fast radiative transitions and RISC processes, which indicate restrained efficiency roll‐offs in devices.^[^ ^31^ ^]^ The reorganization energies were calculated to be 0.11 and 0.14 eV, respectively, for Th‐Cz‐BN3 and Th‐Cz‐BN6 (Figure S28, Supporting Information). The main contributions to reorganization energy for Th‐Cz‐BN3 and Th‐Cz‐BN6 were similar, with high‐frequency stretching vibrations of C─C bonds in phenyl groups between the thiophene and benzazole units and low‐frequency bending vibrations of the whole molecules, as illustrated in Figure S29 (Supporting Information). The main Huang‐Rhys factors (>0.3) resulted from the low‐frequency bending vibrations, which were described in Figure S30 (Supporting Information). The highest Huang‐Rhys factor of 1.01 could be mainly ascribed to the bending vibrations of the alkyl groups, benzothiophene group, and phenyl in 3,6‐di‐tert‐butylcarbazole of Th‐Cz‐BN3. The bending vibrations of the phenyl at the para position of the boron atom and the alkyl groups also contributed to Huang‐Rhys factors of 0.32–0.61 at 23.35–158.25 cm^−1^. Similar bending vibrations were also obtained in Th‐Cz‐BN6. The bending vibrations of the alkyl groups and the hanging phenyl groups contributed to the main Huang‐Rhys factors of 0.32–1.24 at 21.47–51.81 cm^−1^.

### 2.3 Photophysical Properties

**Figure 3:**
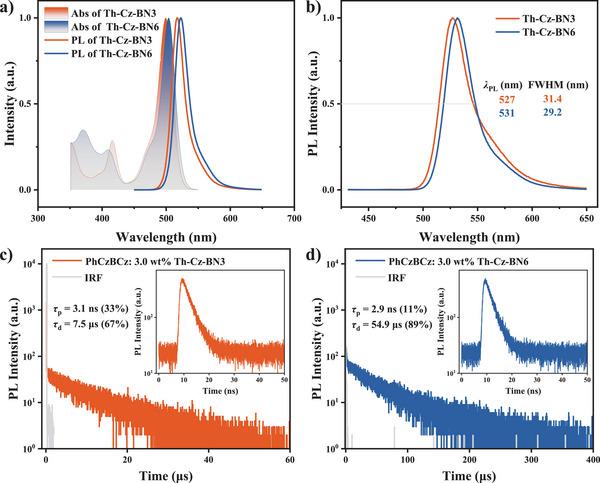
a) Absorption and PL spectra of Th‐Cz‐BN3 and Th‐Cz‐BN6 in solutions (10^−5^ m, toluene). b) PL spectra of Th‐Cz‐BN3 and Th‐Cz‐BN6 in doped films (3.0 wt.% in PhCzBCz). c) Transient PL curves of Th‐Cz‐BN3 in the doped film at room temperature. d) Transient PL curves of Th‐Cz‐BN6 in the doped film at room temperature.

The absorption and photoluminescence (PL) spectra of Th‐Cz‐BN3 and Th‐Cz‐BN6 in dilute solutions (10^−5^ m) are shown in **Figure** **3**a. The sharp absorption peaks at 499 and 504 nm of Th‐Cz‐BN3 and Th‐Cz‐BN6 could be ascribed to the short‐range charge‐transfer. The emission peaks of Th‐Cz‐BN3 and Th‐Cz‐BN6 were 517 and 523 nm, respectively, with FWHM of 24 and 26 nm. The results were consistent with the above‐mentioned theoretical calculation results. The Δ*E*_ST_ values were calculated to be 0.15 and 0.20 eV for Th‐Cz‐BN3 and Th‐Cz‐BN6, based on their PL and phosphorescence spectra in solutions (Figure S31, Supporting Information). It's worth noting that there were two emission peaks of the PL spectra at 77 K of Th‐Cz‐BN3 and Th‐Cz‐BN6 in solution; the short‐wavelength emission peaks could be ascribed to the radiative transition from S_1_ to S_0_, and the long‐wavelength emission peaks could be ascribed to the radiative transition from T_1_ to S_0_, which were consistent with the respective phosphorescence spectra. Figure 3b illustrates the emission spectra of Th‐Cz‐BN3 and Th‐Cz‐BN6 in doped films (host: PhCzBCz, 3.0 wt.%). The emission peaks of the doped films were 527 and 531 nm for Th‐Cz‐BN3 and Th‐Cz‐BN6, respectively, along with FWHM of 31.4 and 29.2 nm, which was opposite to the performance in solutions. The results indicated a more severe aggregation effect in doped films of Th‐Cz‐BN3, which induced more red‐shifted and wider emission spectra.^[^ ^32^ ^]^ The PL intensities of Th‐Cz‐BN3 and Th‐Cz‐BN6 in solutions with different ratios of tetrahydrofuran and water were measured to further verify the aggregation‐caused quenching (ACQ) effect of them, as shown in Figure S32a,b (Supporting Information). The PL intensities decreased sharply when the water ratio exceeded 40%, indicating the ACQ effect. Moreover, obvious emission peaks of Th‐Cz‐BN3 at ≈555 nm were observed, which could be ascribed to the excimer fluorescence. No excimer fluorescence was observed in Th‐Cz‐BN6‐based solutions, which resulted from the twisty configurations and inhibited the generation of the excimer of Th‐Cz‐BN6. An increase in PL intensities of the 60% THF samples was obtained, which could be ascribed to the improved exciton concentrations, and the ACQ effect was not prominent. The absolute photoluminescence quantum yields (PLQYs) of the doped films under N_2_ were measured to be 99.8 ± 0.2% and 99.5 ± 0.1% for Th‐Cz‐BN3 and Th‐Cz‐BN6, respectively. Figure 3c,d demonstrates the transient PL curves of Th‐Cz‐BN3 and Th‐Cz‐BN6‐doped films. The prompt fluorescence lifetime (*τ*_p_) of 3.1 ns and the delayed fluorescence excited state lifetime (*τ*_d_) of 7.5 µs were observed for Th‐Cz‐BN3, which were 2.9 ns and 54.9 µs for the Th‐Cz‐BN6‐based film. Additionally, the Förster radii (*R*_0_) of Th‐Cz‐BN3 and Th‐Cz‐BN6‐based films were calculated to be 27.8–28.8 Å, and the Förster energy transfer (FRET) efficiencies (*E*) were 18.4–23.7%. The distances between PhCzBCz and the emitters were calculated to be 35.1–35.6 Å, and the rates of FRET (*k*_FRET_) were 6.0–8.0 × 10^7^ s^−1^, according to the normalized PL spectrum, transient PL spectrum of PhCzBCz, and absorption spectra of the emitters, as illustrated in Figure S33 (Supporting Information) and summarized in Table S4 (Supporting Information). The TADF nature of Th‐Cz‐BN3 and Th‐Cz‐BN6 was further confirmed by temperature‐dependent transient decay curves, as shown in Figure S34 (Supporting Information).^[^ ^33^ ^]^ The rate constants of the photophysical processes were also calculated and shown in **Table** **1**. Th‐Cz‐BN3 not only exhibited rapid *k*_r,S_ of 10.6 × 10^7^ s^−1^, but also presented rapid *k*_RISC_ of 4.0 × 10^5^ s^−1^, which could be ascribed to the rigid planar configuration, larger oscillator strength, smaller Δ*E*_ST_, and larger SOC constants between S_1_ and T_2_ of Th‐Cz‐BN3. In general, the radiative transition and RISC processes are contradictory. Although the more planar structure of Th‐Cz‐BN3 and a slower *k*_ISC_ were obtained, a more rapid *k*_RISC_ was observed owing to the smaller Δ*E*_ST_ and larger SOC constant of Th‐Cz‐BN3, which contributed to a much shorter delayed fluorescence excited state lifetime. According to Fermi's golden rule, the *k*_RISC_ is proportional to the squared SOC constants and inversely proportional to the squared Δ*E*_ST_ values. A decrease of Δ*E*_ST_ value (0.05 eV) of Th‐Cz‐BN3 would contribute to a 1.8 times increase of *k*_RISC_. However, the *k*_RISC_ of Th‐Cz‐BN3 was 2.4 times higher than that of Th‐Cz‐BN6, so the larger SOC constants of Th‐Cz‐BN3 also contribute to the more rapid *k*_RISC_. It's worth mentioning that although Th‐Cz‐BN6 demonstrated a rather rapid *k*_RISC_ of 1.7 × 10^5^ s^−1^, a much smaller *k_r,S_* ·*K_eq_* was obtained owing to the slow *k*_r,S_, which would contribute to severe efficiency roll‐offs in devices.^[^ ^31^ ^]^

**Table 1:** Photophysical properties of Th‐Cz‐BN3 and Th‐Cz‐BN6.

Compound	*λ*_Abs_ [nm]	*λ*_PL_ [nm]^a)^	FWHM [nm]^b)^	Δ*E*_ST_ [eV]	PLQY [%]	*Ф*_p_ [%]	*Ф*_d_ [%]	*τ*_p_ [ns]	*τ*_d_ [µs]	*k*_r,S_ [× 10^7^ s^−1^]	*k*_ISC_ [× 10^8^ s^−1^]^c)^	*k*_RISC_ [× 10^5^ s^−1^]	*k_r,S_ K_eq_* [×10^4^ s^−1^]^d)^
Th‐Cz‐BN3	499	517/ 527	24/ 31	0.15	99.8 ± 0.2	32.9	66.9	3.1	7.5	10.6	2.2	4.0	14.5
Th‐Cz‐BN6	504	523/ 531	26/ 30	0.20	99.5 ± 0.1	10.9	88.6	2.9	54.9	3.8	3.1	1.7	1.9

^a)^ The emission maximum of the spectra (left: in solutions; right: in doped films);

^b)^ FWHM of the spectra (left: in solutions; right: in doped films);

^c)^ The rate of the intersystem crossing process;

^d)^ The figure of merit for efficiency roll‐off calculated by $k_{r,S}K_{\mathrm{eq}}=\frac{4k_{r,S}k_{\mathrm{RISC}}}{3k_{r,S}+4k_{\mathrm{ISC}}}.$ ^[^ ^31^ ^]^

### 2.4 Electroluminescent Properties

**Figure 4:**
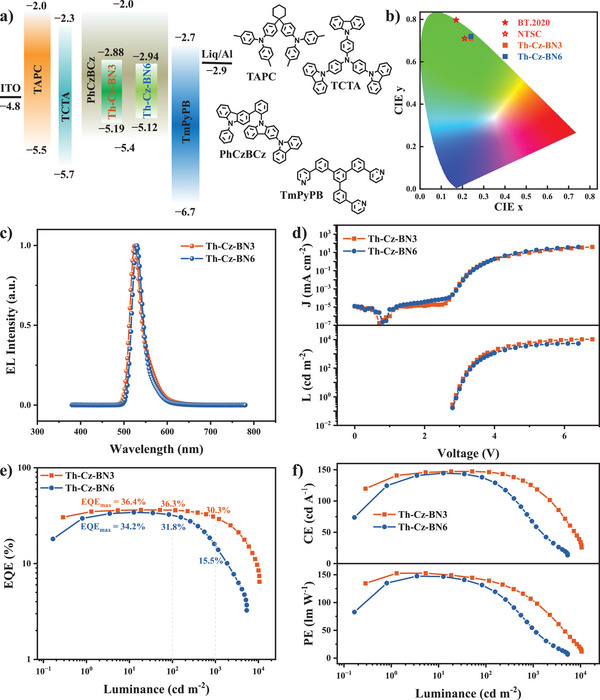
a) Device structures, chemical structures, and energy levels of the materials used in devices. CIE coordinates b), EL spectra c), luminance‐current density‐voltage curves d), EQE‐luminance e), PE‐CE‐luminance f) of the Th‐Cz‐BN3 and Th‐Cz‐BN6‐based devices with the doping concentration of 3.0 wt.%.

**Figure 5:**
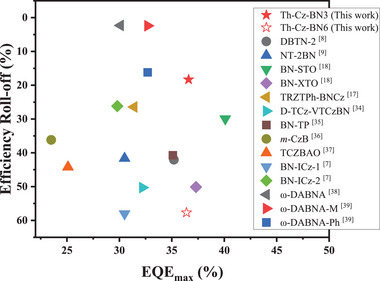
A comparison of the maximum EQE and efficiency roll‐off at 1000 cd m^−2^ of MR‐TADF emitter‐based non‐sensitized devices with CIEy over 0.7.

The electroluminescent properties of Th‐Cz‐BN3 and Th‐Cz‐BN6 were represented by fabricating OLED devices with structures of [ITO (45 nm)/TAPC (70 nm)/TCTA (5 nm)/PhCzBCz:x wt.% emitters (25 nm)/TmPyPB (40 nm)/Liq (2 nm)/Al (100 nm)]. The device structures, chemical structures, and energy levels of the materials used in devices are illustrated in **Figure** **4**a. TAPC, TCTA, TmPyPB, and Liq were employed as the hole‐transporting, electron‐blocking, electron‐transporting, and electron‐injecting materials, respectively. PhCzBCz was the host. ITO and Al were used as the anode and cathode, respectively. The optimal doping concentrations were explored by varying the doping concentrations with 1.0, 3.0, 5.0, and 10.0 wt.%. The EL properties of the devices with different doping concentrations are illustrated in Figures S35 and S36 (Supporting Information) and summarized in **Table** **2**. The turn‐on voltages of the devices were ≈3.0 V. As expected, Th‐Cz‐NB3 exhibited more concentration‐dependent EL spectra with peaks ranging from 522 to 529 nm, along with FWHM ranging from 28 to 50 nm when increasing the doping concentrations from 1.0 to 10.0 wt.%. While Th‐Cz‐BN6 presented more concentration‐independent EL spectra with peaks ranging from 528 to 534 nm and FWHM ranging from 28 to 32 nm, which could be ascribed to the more twisted configuration and weakened intermolecular interactions.^[^ ^34^ ^]^ Nevertheless, all the devices exhibited high maximum external quantum efficiencies (EQEs) over 31.6%, and the optical doping concentrations of Th‐Cz‐BN3 and Th‐Cz‐BN6‐based devices were 3.0 wt.%. The detailed EL properties and data are shown in Figure 4b–f. The Th‐Cz‐BN3‐based device exhibited emission peaks at 525 nm with an FWHM of 31 nm and CIE coordinates of (0.24, 0.71). The maximum current efficiency (CE), power efficiency (PE), and EQE were 147.3 cd A^−1^, 152.7 lm W^−1^, and 36.4% of the Th‐Cz‐BN3‐based device, respectively. The device of Th‐Cz‐BN6 demonstrated an EL spectrum with peaks at 530 nm, FWHM of 29 nm, and pure green CIE coordinates of (0.24, 0.72). It's worth mentioning that the Th‐Cz‐BN3‐based non‐sensitized device exhibited very small efficiency roll‐offs of 0.3% and 16.8% at 100 and 1000 cd m^−2^, respectively. The results were much gentler than those of the Th‐Cz‐BN6‐based non‐sensitized device, which were 7.0% and 54.7% at 100 and 1000 cd m^−2^, respectively. Further, the Th‐Cz‐BN3 and Th‐Cz‐BN6‐based films exhibited similar horizontal dipole ratios ≈84% (Figures S37 and S38, Supporting Information), indicating similar light out‐coupling efficiencies of Th‐Cz‐BN3 and Th‐Cz‐BN6‐based devices. Moreover, the angle‐dependent EL spectra of the devices were also measured. As shown in Figure S39 (Supporting Information), the EL intensity of the spectra at different angles followed the Lambertian pattern, indicating the accuracy of the EQE values. As displayed under the photoexcitation, the lifetime of the delayed fluorescence excited state of the Th‐Cz‐BN3‐based device (16.8 µs) was much shorter than that of the Th‐Cz‐BN6‐based one (119.7 µs), obtained from the transient EL spectra (Figure S40, Supporting Information). So, the significantly suppressed efficiency roll‐offs of the Th‐Cz‐BN3‐based device could be ascribed to the rapid *k*_r,S_, and *k*_RISC_ of Th‐Cz‐BN3. The results are better than most reported pure green MR‐TADF emitter‐based non‐sensitized OLED devices with CIEy over 0.7, as illustrated in **Figure** **5** and summarized in Table S5 (Supporting Information).^[^ ^7^, ^8^, ^9^, ^17^, ^18^, ^35^, ^36^, ^37^, ^38^, ^39^, ^40^ ^]^

**Table 2:** EL properties of the devices.

Emitters	*V*_on_ [V]^a)^	*λ*_em_ [nm]	FWHM [nm]	CE^b)^ [cd A^−1^]	PE^b)^ [lm W^−1^]	EQE^b)^ [%]	CIE(x,y)^c)^
1.0 wt.% Th‐Cz‐BN3	3.0	522	28	134.9, 128.8, 92.0	139.4, 122.1, 77.0	34.8, 33.1, 13.7	(0.19, 0.73)
3.0 wt.% Th‐Cz‐BN3	2.9	525	31	147.3, 146.6, 123.1	152.7, 140.7, 103.5	36.4, 36.3, 30.3	(0.24, 0.71)
5.0 wt.% Th‐Cz‐BN3	2.9	527	36	142.2, 140.5, 118.0	151.6, 135.5, 98.5	34.7, 34.2, 28.7	(0.28, 0.68)
10.0 wt.% Th‐Cz‐BN3	2.8	529	50	136.7, 133.3, 106.8	143.1, 128.8, 87.2	33.3, 32.4, 25.7	(0.32, 0.66)
1.0 wt.% Th‐Cz‐BN6	3.0	528	28	134.6, 119.3, 48.3	136.4, 111.9, 38.0	32.3, 28.4, 11.6	(0.23, 0.73)
3.0 wt.% Th‐Cz‐BN6	3.0	530	29	144.6, 136.0, 65.3,	147.4, 128.6, 52.3	34.2, 31.8, 15.5	(0.24, 0.72)
5.0 wt.% Th‐Cz‐BN6	2.9	533	31	140.2, 132.6, 64.8	148.3, 125.5, 50.9	32.6, 30.8, 15.0	(0.26, 0.71)
10.0 wt.% Th‐Cz‐BN6	2.8	534	32	136.2, 124.6, 52.9	146.3, 119.3, 40.5	31.6, 28.8, 11.9	(0.28, 0.70)

^a)^ The turn‐on voltage of the devices at ≈1.0 cd m^−2^;

^b)^ The maximum and the values at 100 and 1000 cd m^−2^;

^c)^ Record at ≈1000 cd m^−2^.

Owing to the instability of TAPC and PhCzBCz, the actual operational stability of Th‐Cz‐BN3 and Th‐Cz‐BN6 was measured via a more stable device structure, including [ITO/BPBPA (50 nm)/Prime (10 nm)/GH: 3.0 wt.% ThCz‐BN3 or ThCz‐BN6 (40 nm)/TRZ‐DBF (5 nm)/NA‐An‐Im (35 nm)/Liq (2 nm)/Al (100 nm)]. The device structure is illustrated in Figure S41 (Supporting Information).^[^ ^41^ ^]^ The EL properties of the devices are shown in Figure S42 and Table S6 (Supporting Information). The EL emission peaks were slightly red‐shifted, which could be ascribed to the stronger polar host matrix or the different optical microcavity effect resulting from the different device structures. The operational LT_95_ (lifetime to 95% of the initial luminance) of the devices were 50.0 and 239.1 h at 1000 cd m^−2^ for Th‐Cz‐BN3 and Th‐Cz‐BN6‐based devices, respectively, as shown in Figure S43 (Supporting Information). The further suppressed efficiency roll‐offs of the devices could be ascribed to the compressed delayed fluorescence lifetime, as illustrated in Figure S44 (Supporting Information). To further optimize the device performance, phosphor‐sensitized devices were also fabricated, and the EL properties are shown in Figure S45 (Supporting Information) and summarized in Table S6 (Supporting Information). The turn‐on voltages of the devices were 2.2 V. The EL emission peaks were 529 and 533 nm of the Th‐Cz‐BN3 and Th‐Cz‐BN6‐based devices, along with FWHM of 31 and 30 nm, respectively. The maximum CE, PE, and EQE of the Th‐Cz‐BN3 and Th‐Cz‐BN6‐based devices were 112.0 cd A^−1^, 159.9 lm W^−1^, 27.4%, and 115.9 cd A^−1^, 165.5 lm W^−1^, 27.4%, respectively. The efficiency roll‐offs were also much controlled, which were 5.8% and 19.7% at 1000 and 10000 cd m^−2^ of the Th‐Cz‐BN3‐based device, respectively, which were also better than those of the Th‐Cz‐BN6‐based device. It's worth mentioning that the maximum EQEs of the sensitized devices were lower than those of the PhCzBCz‐based non‐sensitized ones, which could be ascribed to the lower horizontal dipole ratios of the sensitized films, as shown in Figures S46 and S47 (Supporting Information), in consideration of the similar PLQYs values of the sensitized films for Th‐Cz‐BN3 (99.6 ± 0.2%) and Th‐Cz‐BN6 (99.4 ± 0.2%). The results indicated the influence on the horizontal dipole ratios of the host matrix. The devices exhibited long‐lifetime operational LT_95_ of 39.0 and 181.1 h at a current density of 10 mA cm^−2^ (Figure S48, Supporting Information), which could be calculated to be 1083.7 and 5895.2 h with an initial luminance of 1000 cd m^−2^ and a degradation acceleration factor of 1.75, indicating their industrial potential. In addition, we have explored the difference in operational lifetimes of the two emitters from the stability of device physics and the materials themselves. First, the inferior performance of Th‐Cz‐BN3 would not be ascribed to the high energy levels of excitons resulting from the triplet‐triplet annihilation (TTA). The Th‐Cz‐BN3‐based devices also demonstrated shorter transient EL lifetime than that of the Th‐Cz‐BN6‐based one (Figure S49, Supporting Information). Moreover, there was no obvious charge‐carrier trapping from the transient EL spectra. On the other hand, photostability experiments were performed with a continuous excitation light source of 365 nm, and the time‐resolved emission spectra for the doped films were recorded (Figure S50, Supporting Information). A 23% luminance intensity decay was obtained for Th‐Cz‐BN3‐based films, and a 30% luminance intensity decay was obtained for Th‐Cz‐BN6‐based films, indicating that Th‐Cz‐BN3 was more stable than Th‐Cz‐BN6 under photostimulation. Furthermore, 20‐cycle CV curves were performed to test the electrochemical stability of Th‐Cz‐BN3 and Th‐Cz‐BN6. As illustrated in Figure S51 (Supporting Information), reversible CV curves were obtained, which indicates the electrochemical stability of the emitters. Moreover, the purity of Th‐Cz‐BN3 and Th‐Cz‐BN6 was tested by high‐performance liquid chromatography (HPLC), and the purity of Th‐Cz‐BN3 and Th‐Cz‐BN6 was 98.6% and 99.7% (Figures S52 and S53, Supporting Information), respectively. The impurities of Th‐Cz‐BN3 would contribute to more defect states, which may impact the operational lifetime significantly.^[^ ^42^ ^]^ Even so, the factors influencing the device's operational lifetime were complex, and we can't provide an exact reason for the operational lifetime difference between the two emitters. Further exploration is being performed in our laboratory, and the results will be published in our future work.

## 3 Conclusion

In conclusion, two pure green MR‐TADF emitters have been proposed by inserting sulfur atoms connecting with the 3‐position carbon atoms of the carbazole derivatives, which improved the HOMO energy levels and achieved pure green emission. Th‐Cz‐BN3 and Th‐Cz‐BN6 were analogous isomers. Even so, the large difference in configurations of Th‐Cz‐BN3 and Th‐Cz‐BN6 contributed to a significant difference in photophysical and electroluminescent properties. Eventually, the Th‐Cz‐BN3‐based non‐sensitized device presented a maximum EQE of 36.4% and a remarkable efficiency roll‐off of 16.8% at 1000 cd m^−2^, which further proved the importance of *k*_r,S_ in regulating the device efficiency roll‐offs. Moreover, Th‐Cz‐BN3 and Th‐Cz‐BN6‐based devices showed reasonable operational lifetimes. The results revealed that the sulfur‐doped positions in MR‐TADF emitters significantly influence the energy levels, photophysical, and electroluminescent properties of the emitters and paved the way for designing highly efficient pure green MR‐TADF emitters with small efficiency roll‐offs.

## 4 Experimental Section

[CCDC 2452814 (for Th‐Cz‐BN3) and 2449214 (for Th‐Cz‐BN6) contain the supplementary crystallographic data for this paper. These data can be obtained free of charge from The Cambridge Crystallographic Data Centre via www.ccdc.cam.ac.uk/data_request/cif.]

## Conflict of Interest

The authors declare no conflict of interest.

## Supporting information



Supporting Information

## Data Availability

Research data are not shared.
