# Supplementary material for: Sulfur‐Embedded Pure Green Multiple Resonance TADF Emitters: Optimizing Photophysical and Electroluminescent Properties
Source: Adv Sci (Weinh). 2025 Oct 29;13(3):e12796. doi: 10.1002/advs.202512796 (PMC12806378; doi:10.1002/advs.202512796)
Supplement: Supplementary file 1 — Supporting Information [file ADVS-13-e12796-s001.docx]

Supporting Information

Sulfur-Embedded Pure Green Multiple Resonance TADF Emitters: Optimizing Photophysical and Electroluminescent Properties

Chao Jiang,^#^ Yufang Nie,^#^ Chi Cao, Xiaoxian Song, Jie Liang, Xuming Zhuang, Zhiqiang Li, Baoyan Liang*, and Yue Wang*

Materials and methods

General information

Thermo scientific ISQ 7000 mass spectrometers were employed to measure the mass spectra. Bruker AVANCE III 400 and 600 MHz spectrometers were selected to measure the ^1^H and ^13^C NMR spectra with tetramethylsilane (TMS) as the internal standard. Shimadzu RF-6000 spectrometer and Shimadzu UV-2600i spectrophotometer were adopted to record the PL emission spectra in solution and UV-Vis absorption, respectively. The fluorescence and phosphorescence spectra taken at liquid nitrogen temperature (77 K) were recorded by Edinburgh FLS1000, as well as the transient PL decay. The photoluminescence quantum yields (PLQYs) of organic films were detected from Hamamatsu Quantaurus-QY, an absolute quantum yield spectrometer, with the excitation wavelength of 365 nm. The angle-dependent spectra of the films were measured by the R1-OLED from Ffuxiang Optical Co., Ltd. In the range of 25 to 900 °C, TA TGA 5500 thermogravimeter was selected to perform the thermogravimetric analysis (TGA) under a nitrogen atmosphere at a heating rate of 10 K min^-1^. TA DSC 250 was employed to measure glass transition temperature, melting point, and crystallization temperature. Ivium CompactStat.h electrochemical workstation was used to measure the electrochemical property with a glassy carbon electrode as the working electrode, platinum wire as the auxiliary electrode, a porous glass wick Ag/Ag^+^ as the pseudo reference electrode, and ferrocene/ferrocenium as the internal standard. 0.1 M solution of n-Bu_4_NPF_6_ was the supporting electrolyte utilized to measure the oxidation (in anhydrous dichloromethane) or reduction (in anhydrous tetrahydrofuran) potentials at a scan rate of 100 mV s^–1^. The end products were sublimated with a BOF-5-50 vacuum sublimation instrument (Anhui BEQ Equipment Technology Co., Ltd) before device fabrication.

Theoretical calculation methods

The ground state geometries were fully optimized by the B3LYP method, including Grimme’s dispersion correction with 6-31G (d,p) basis set using the Gaussian 09 software package.^[S1-3]^ HOMO and LUMO were visualized with Multiwfn software package.^[S4]^ The excited state properties were calculated by TDDFT with the same theory level as DFT. The spin-orbit coupling matrix elements were performed in ORCA 4.2.1 software.^[S5]^

OLED fabrication and characterization

The indium tin oxide (ITO) glass substrates with a sheet resistance of 35 Ω per square was cleaned with optical detergent, deionized water, acetone, and isopropanol successively. After UV-ozone treatment for 15 min, the substrates were transferred to a vacuum deposition system. Organic materials and Al layers were consecutively thermally evaporated onto the substrates at rates of 1 Å s^-1^ and 10 Å s^-1^, respectively, in a vacuum chamber of < 9 × 10^−5^ Pa. The EL spectrum, CIE coordinate, and luminance intensity of the OLEDs were recorded by Konica Minolta CS2000, meanwhile, the current density (J) and driving voltage (V) were recorded by Keithley 2400. By assuming Lambertian distribution, the external quantum efficiency (EQE) was estimated according to brightness, electroluminescence spectrum, and current density. Device operational stability was measured by using a multichannel device lifetime system cooperated with a silicon photodiode and digital multimeter to measure the parameters of device stability at a constant current density of 10 mA cm^-2^, and devices were measured at a room temperature of 23 ± 1 °C. The materials employed in device fabrication were all purchased.

Analysis of Rate Constants

$$k_{p}=\frac{1}{\tau_{p}}$$

$$k_{d}=\frac{1}{\tau_{d}}$$

$$k_{r,S}=\frac{\varphi_{p}}{\tau_{p}}$$

$$k_{nr,S}=\frac{1-\varphi_{PL}}{\varphi_{PL}}k_{r,S}$$

$$k_{ISC}=k_{p}-k_{r,S}-k_{nr,S}$$

$$k_{RISC}=（k_{p}k_{d}\varphi_{d}/k_{ISC}\varphi_{p}）$$

The photophysical rate constants of the films could be estimated using Formula (1).^[S6]^ *τ*_p_ and *τ*_d_ are the prompt and delayed lifetimes, *k*_p_ and *k*_d_ represent the decay rate constants for prompt and delayed fluorescence, respectively. *φ*_p_ and *φ*_d_ indicate prompt and delayed fluorescence quantum yields. *φ*_PL_ indicates the total PLQY. *k*_r,S and_ *k*_nr,S_ are rates of radiative transition and nonradiative transition of S_1_, respectively. *k*_ISC_ and *k*_RISC_ are the rate constants of intersystem crossing and reverse intersystem crossing.

Calculation of Förster radius (*R*_0_)

$$R_{0}^{6}=\frac{9000\cdot(ln10)\cdot k^{2}\cdot\varphi_{\mathrm{PLQY}}\cdot J}{128\pi^{5}n^{4}N_{A}}$$

The Förster radius is calculated using the equation, in which k^2^ is the orientation factor. φ_PLQY_ is the PLQY of the donor. *J* is the spectral overlap integral. *n* is the refractive index of the medium. *N*_A_ is the avogadro's number.

Calculation of the efficiency of FRET (E)

$$E=1-\frac{\tau_{DA}}{\tau_{D}}$$

τ_DA_ is the lifetime of the prompt fluorescence of the host-guest film, and τ_D_ is the lifetime of the prompt fluorescence of the host film.

Calculation of the rate of FRET (*k*_FRET_)

$$E=\frac{1}{1+{(r/R_{0})}^{6}}$$

$$k_{\mathrm{FRET}}=\frac{1}{\tau_{D}}{(\frac{R_{0}}{r})}^{6}$$

*r* is the distance of the host and guest.

**Figure S1.** Theoretical calculation results of the benzothienocarbazole groups.

Synthesis of the compounds

Scheme S1. The synthetic routes of Th-Cz-BN3 and Th-Cz-BN6.

**Synthesis of 1-(5,5,8,8-tetramethyl-3-nitro-5,6,7,8-tetrahydronaphthalen-2-yl)dibenzo[*b*,*d*]thiophene (****Th-Cz-BN3-A):** Dibenzo[*b*,*d*]thiophen-1-ylboronic acid (5.47 g, 24.0 mmol), 6-bromo-1,1,4,4-tetramethyl-7-nitro-1,2,3,4-tetrahydronaphthalene (6.24 g, 20.0 mmol), K₂CO₃ (5.52 g, 40.0 mmol), and Pd(PPh₃)₄ (1.15 g, 1.0 mmol) were dissolved in a mixed solvent of Tol/ EtOH/ H₂O (80.0/ 20.0/ 20.0 mL). The mixture was heated to 110°C under a nitrogen atmosphere and stirred for 8 hours. After the reaction was completed, the mixture was cooled to room temperature and extracted with dichloromethane and water. The organic phase was concentrated and purified by column chromatography, The target molecule was obtained as a yellow solid of 6.41 g with a yield of 79%. EI-MS (m/z): [M^+^] calcd for C_26_H_25_NO_2_S, 415.16, found, 415.20, ^1^H NMR (500 MHz, CDCl_3_) *δ* 8.19 (s, 1H), 7.93 (d, *J* = 8.0 Hz, 1H), 7.86 (d, *J* = 7.9 Hz, 1H), 7.50 (t, *J* = 7.7 Hz, 1H), 7.43 (s, 1H), 7.37 (t, *J* = 7.6 Hz, 1H), 7.22 (d, *J* = 7.3 Hz, 1H), 7.11 (t, *J* = 7.6 Hz, 1H), 6.89 (d, *J* = 8.2 Hz, 1H), 1.91 – 1.79 (m, 4H), 1.52 (s, 3H), 1.47 (s, 3H), 1.32 (s, 3H), 1.25 (s, 3H).

Figure S2. Mass spectrum of **Th-Cz-BN3-A.**


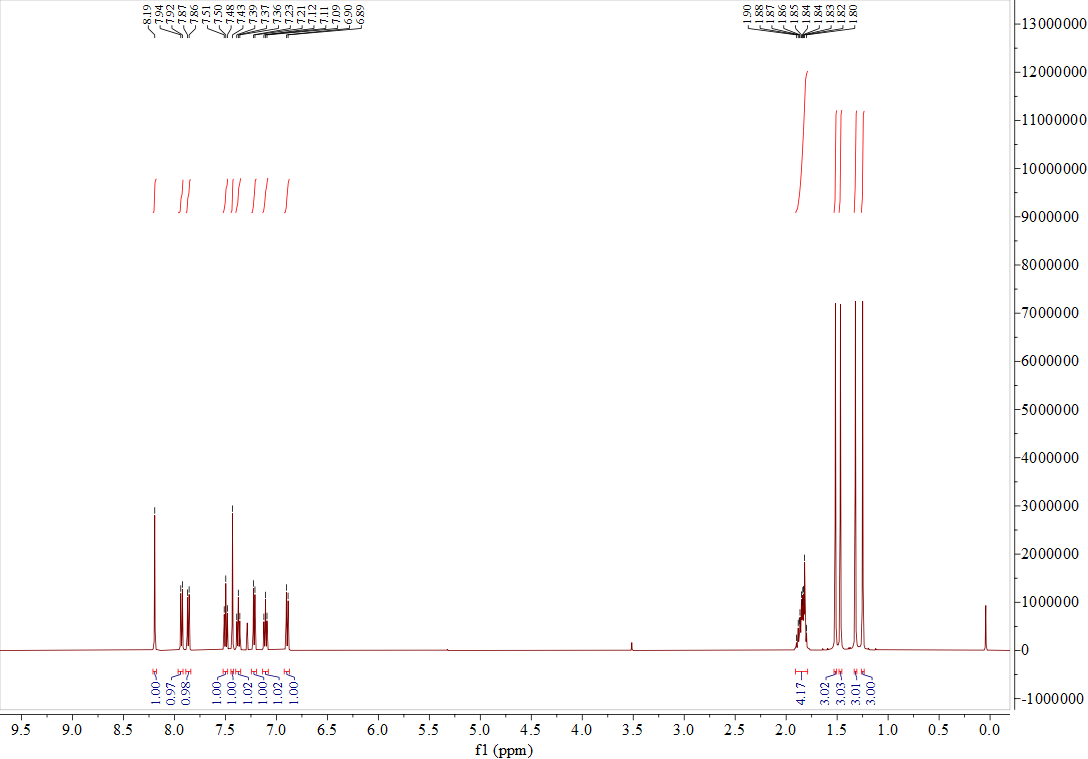


Figure S3. ^1^H NMR spectrum of **Th-Cz-BN3-A.**

**Synthesis of 10,10,13,13-tetramethyl-10,11,12,13-tetrahydro-8*H*-benzo[*b*]benzo-[4,5]thieno[3,2-*g*]carbazole (Th-Cz-BN3-B):** Th-Cz-BN3-A (6.41 g, 15.8 mmol) and PPh₃ (41.40 g, 158 mmol) were dissolved in 100 mL of o-dichlorobenzene. The mixture was heated to 180°C under a nitrogen atmosphere with stirring for 12 hours. After the reaction was complete and cooled to room temperature, the solvent was removed under reduced pressure. Purification by column chromatography yielded 3.03 g of a white solid in 50.0% yield. EI-MS (m/z): [M^+^] calcd for C_26_H_25_NS, 383.17, found, 383.14, ^1^H NMR (600 MHz, CDCl_3_) *δ* 9.17 (d, *J* = 8.0 Hz, 1H), 8.84 (s, 1H), 8.19 (s, 1H), 7.98 (d, *J* = 7.3 Hz, 1H), 7.83 (d, *J* = 8.4 Hz, 1H), 7.62 (t, *J* = 7.5 Hz, 1H), 7.55 (d, *J* = 8.4 Hz, 1H), 7.52 (t, *J* = 7.4 Hz, 1H), 7.49 (s, 1H), 1.86 - 1.81 (m, 4H), 1.52 (s, 6H), 1.43 (s, 6H).

Figure S4. Mass spectrum of **Th-Cz-BN3-B**.


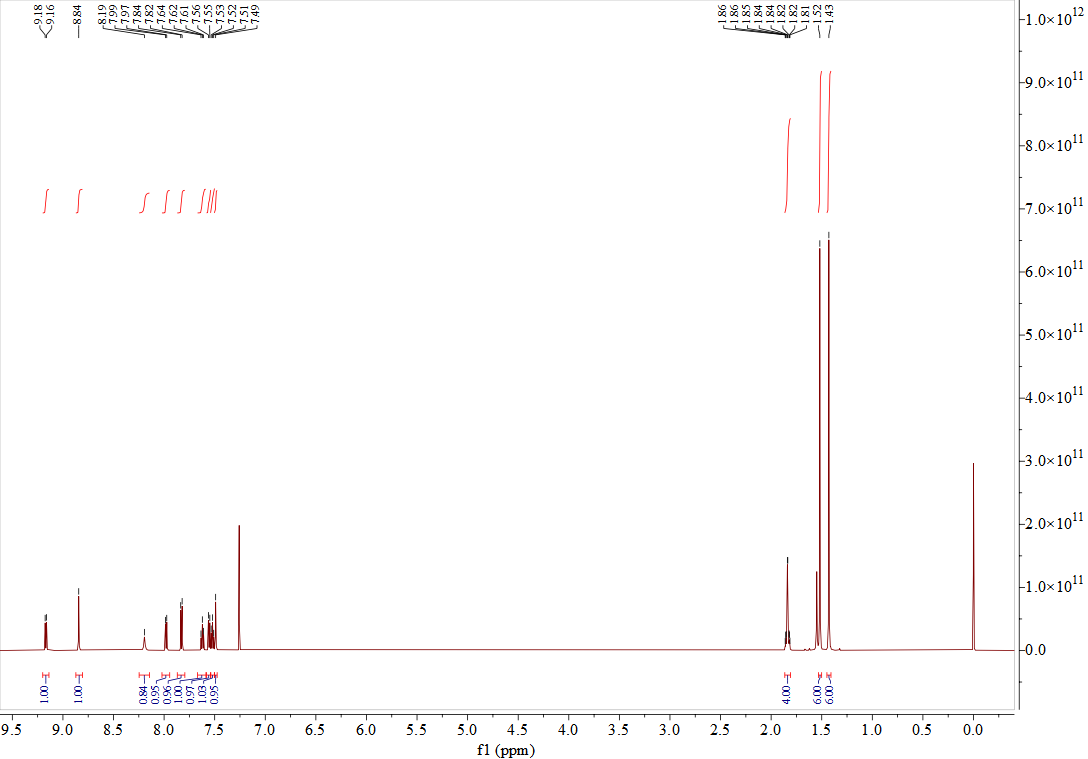


Figure S5. ^1^H NMR spectrum of **Th-Cz-BN3-B**.

Synthesis of 8-(4-chloro-5-(3,6-di-*tert*-butyl-9*H*-carbazol-9-yl)-[1,1'-biphenyl]-yl)-10,10,13,13-tetramethyl-10,11,12,13-tetrahydro-8*H*-benzo[*b*]benzo[4,5]thieno[3,2-*g*]carbazole (Th-Cz-BN3-C): Th-Cz-BN3-C (3.03 g, 7.9 mmol), 3,6-di-*tert*-butyl-9-(4-chloro-5-fluoro-[1,1'-biphenyl]-3-yl)-9*H*-carbazole (3.82 g, 7.9 mmol), and Cs₂CO₃ (5.14 g, 15.8 mmol) were dissolved in 30 mL DMF. The mixture was heated to 150°C under a nitrogen atmosphere and stirred for 8 hours. After the reaction was complete, the mixture was cooled to room temperature, poured into an ammonium chloride aqueous solution, and filtered to collect the filter cake. The crude product was recrystallized with dichloromethane/methanol, The target molecule was obtained as a white solid of 5.69 g with a yield of 85%. EI-MS (m/z): [M+] calcd for C_58_H_55_ClN_2_S, 846.38, found, 846.09.^1^H NMR (600 MHz, CDCl_3_) *δ* 9.23 (d, *J* = 8.1 Hz, 1H), 8.95 (s, 1H), 8.19 (d, *J* = 1.9 Hz, 2H), 7.99 (q, *J* = 3.2 Hz, 3H), 7.89 (d, *J* = 8.5 Hz, 1H), 7.65 (d, *J* = 8.0 Hz, 3H), 7.58 - 7.53 (m, 2H), 7.51 (d, *J* = 8.5 Hz, 1H), 7.48 - 7.43 (m, 3H), 7.40 (t, *J* = 7.4 Hz, 1H), 7.31 (d, *J* = 8.2 Hz, 2H), 7.27 (d, *J* = 8.7 Hz, 1H), 1.86 (d, *J* = 5.4 Hz, 4H), 1.56 (d, *J* = 26.6 Hz, 7H), 1.48 (s, 18H), 1.45 (d, *J* = 4.2 Hz, 6H).

Figure S6. Mass spectrum of **Th-Cz-BN3-C**.


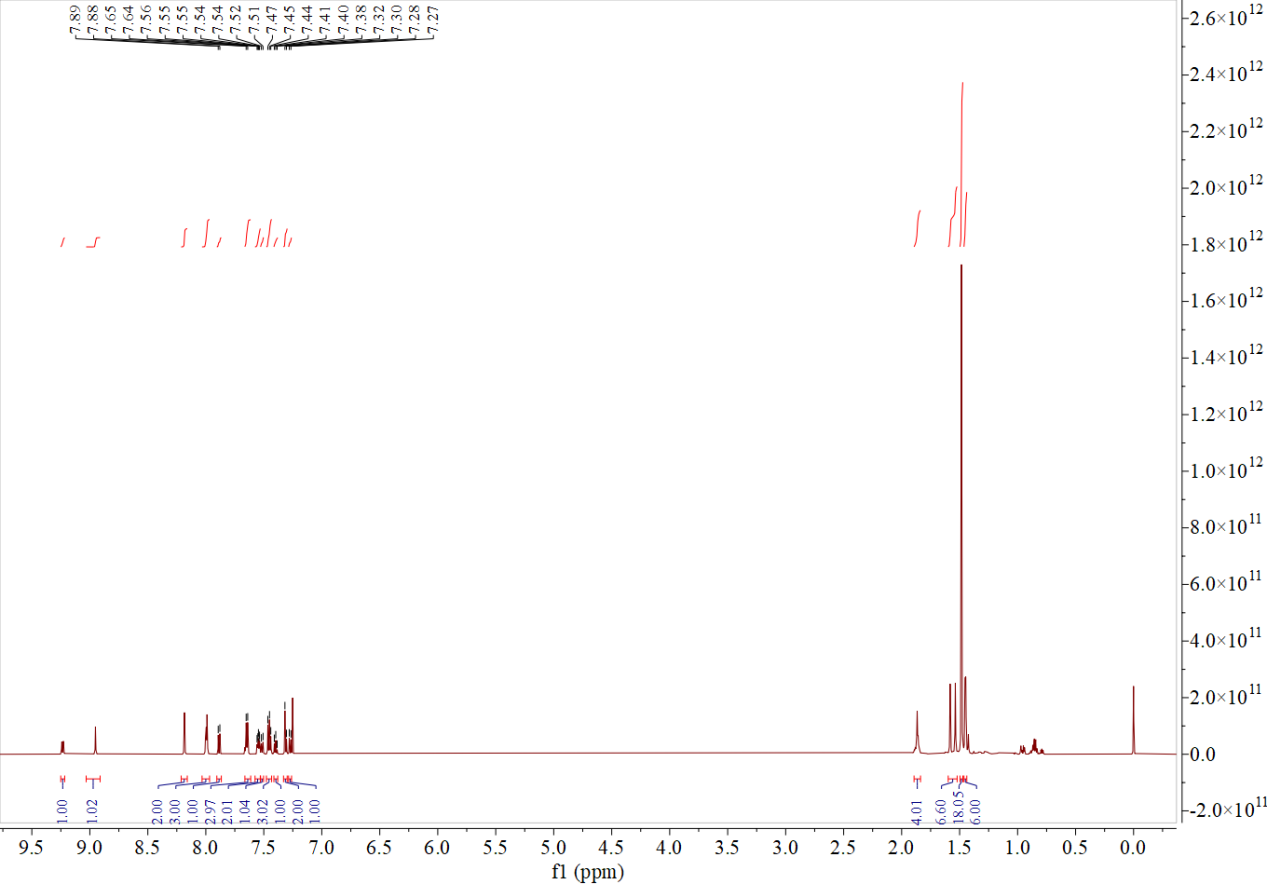


Figure S7. ^1^H NMR spectrum of **Th-Cz-BN3-C**..

**Synthesis of Th-Cz-BN3: Th-Cz-BN3-C** (4.24 g，5.0 mmol) was dissolved in 50.0 mL of tert-butylbenzene. Under nitrogen atmosphere, *t*-BuLi (1.3 M, 7.7 mL, 10.0 mmol) was added dropwise at -78℃ and stirred for 0.5 h, the temperature was raised to 70℃ and stirred for 2 hours. The reaction was then cooled to -40℃, and BBr_3_ (2.50 g, 10.0 mmol) was added dropwise. The mixture was allowed to warm to room temperature and stirred for 1 hour. Subsequently, *N*,*N*-diisopropylethylamine ( 1.29 g, 10.0 mmol) was added dropwise at 0℃, and the reaction system was heated to 150℃ with stirring for 6 hours.Upon completion of the reaction, the mixture was quenched by adding 5.0 mL each of methanol and water. The reaction mixture was extracted with dichloromethane and water. The organic phase was concentrated under reduced pressure and purified by column chromatography, The target molecule was obtained as a yellow solid of 1.27 g with a yield of 31.0%. EI-MS (m/z): [M^+^] calcd for C_58_H_53_BN_2_S, 820.40, found, 820.52.^1^H NMR (600 MHz, CDCl_3_) *δ* 9.28 (s, 1H), 9.23 (d, *J* = 7.8 Hz, 1H), 9.02 (d, *J* = 2.2 Hz, 1H), 8.99 (s, 1H), 8.69 (s, 1H), 8.58 (s, 1H), 8.53 (s, 1H), 8.47 (d, *J* = 2.0 Hz, 1H), 8.42 (d, *J* = 8.7 Hz, 1H), 8.27 (d, *J* = 2.2 Hz, 1H), 8.06 (d, *J* = 7.6 Hz, 1H), 7.98 (d, *J* = 7.4 Hz, 2H), 7.71 - 7.65 (m, 2H), 7.63 (t, *J* = 7.6 Hz, 3H), 7.53 (t, *J* = 7.4 Hz, 1H), 1.91 (s, 4H), 1.69 (s, 9H), 1.56 (d, *J* = 5.4 Hz, 12H), 1.55 (s, 9H).^13^C NMR (151 MHz, CDCl_3_) *δ* 145.53, 145.32, 144.71, 144.30, 144.10, 144.05, 142.00, 141.65, 141.33, 139.14, 138.18, 138.13, 135.76, 134.27, 132.90, 129.32, 129.16, 128.32, 127.57, 127.14, 127.02, 126.74, 125.58, 124.56, 124.46, 123.82, 123.67, 123.25, 121.56, 120.80, 118.95, 117.32, 114.26, 111.59, 107.72, 106.76, 35.33, 35.30, 35.28, 35.18, 34.85, 34.74, 32.77, 32.69, 32.28, 31.88.

Figure S8. Mass spectrum of **Th-Cz-BN3**.

Figure S9. ^1^H NMR spectrum of **Th-Cz-BN3**.


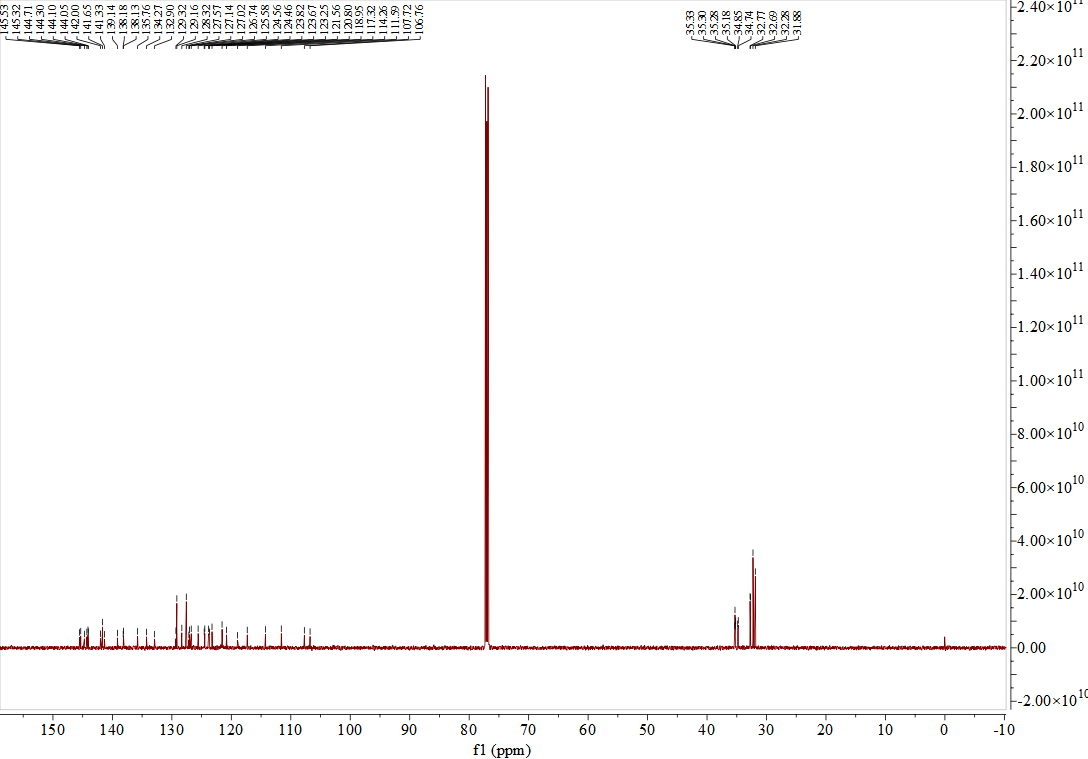


Figure S10. ^13^C NMR spectrum of **Th-Cz-BN3**.

**Synthesis of 4,4,5,5-tetramethyl-2-(4-phenyldibenzo[*b*,*d*]thiophen-3-yl)-1,3,2-dioxaborolane (Th-Cz-BN6-A):** 3-bromo-4-phenyldibenzo[*b*,*d*]thiophene (8.14 g, 24.0 mmol), Bpin_2_ (9.14 g, 36.0 mmol), KOAc(23.52 g, 240.0 mmol) and Pd(dppf)Cl_2_ (0.88 g, 1.2 mmol) were dissolved in 100.0 mL 1,4-Dioxane.The mixture was heated to 120°C under a nitrogen atmosphere with stirring for 12 hours. After the reaction was complete and cooled to room temperature, the mixture was extracted with dichloromethane and water. The organic phase was concentrated under reduced pressure and purified by column chromatography. The target molecule was obtained as a white solid of 6.30 g with a yield of 68.0%.EI-MS (m/z): [M^+^] calcd for Chemical Formula: C_24_H_23_BO_2_S, 386.15, found, 386.19. ^1^H NMR (600 MHz, Chloroform-*d*) *δ* 8.22 - 8.18 (m, 1H), 8.13 (d, *J* = 7.9 Hz, 1H), 7.85 (d, *J* = 7.9 Hz, 1H), 7.80 - 7.76 (m, 1H), 7.53 - 7.50 (m, 2H), 7.48 - 7.40 (m, 5H), 1.18 (s, 12H).

Figure S11. Mass spectrum of **Th-Cz-BN6-A**.

Figure S12. ^1^H NMR spectrum of **Th-Cz-BN6-A**.

Synthesis of 4-phenyl-3-(5,5,8,8-tetramethyl-3-nitro-5,6,7,8-tetrahydronaphthalen-2-yl)dibenzo[*b*,*d*]thiophene (Th-Cz-BN6-B): Th-Cz-BN6-A (6.18g, 16.0 mmol), 6-bromo-1,1,4,4-tetramethyl-7-nitro-1,2,3,4-tetrahydronaphthalene (5.97 g, 19.2 mmol), K₂CO₃ (4.422 g, 32 mmol), and Pd(PPh₃)₄ (0.92 g, 0.8 mmol) were dissolved in a mixed solvent of Tol/ EtOH/ H₂O (80.0/ 20.0/ 20.0 mL). The mixture was heated to 110°C under a nitrogen atmosphere and stirred for 8 hours. After the reaction was complete, the mixture was cooled to room temperature and extracted with dichloromethane and water. The organic phase was concentrated and purified by column chromatography, The target molecule was obtained as a yellow solid of 5.11 g with a yield of 65.0 %. EI-MS (m/z): [M^+^] calcd for Chemical Formula: C_32_H_29_NO_2_S, 491.19, found, 491.25. ^1^H NMR (500 MHz, Methylene Chloride-*d*_2_) δ 8.27 – 8.22 (m, 1H), 8.19 (d, *J* = 8.1 Hz, 1H), 7.83 (dd, *J* = 6.9, 1.9 Hz, 1H), 7.80 (s, 1H), 7.57 – 7.23 (m, 8H), 7.08 (s, 1H), 1.66 (q, *J* = 7.0 Hz, 4H), 1.37 – 1.24 (m, 6H), 1.19 (s, 3H), 0.89 (s, 3H).

Figure S13. Mass spectrum of **Th-Cz-BN6-B**.

Figure S14. ^1^H NMR spectrum of **Th-Cz-BN6-B**.

Synthesis of 8,8,11,11-tetramethyl-6-phenyl-9,10,11,13-tetrahydro-8*H*-benzo[*b*]benzo[4,5]thieno[2,3-*h*]carbazole (Th-Cz-BN6-C): Th-Cz-BN6-B (5.10 g, 10.4 mmol) and PPh_3_ (27.25 g, 104 mmol) was dissolved in 50 mL of *o*-dichlorobenzene, heated to 180°C under a nitrogen atmosphere, and stirred for 12 hours. After the reaction was complete and cooled to room temperature, the mixture was concentrated under reduced pressure and purified by column chromatography. The target molecule was obtained as a white solid of 2.72 g with a yield of 57%. EI-MS (m/z): [M^+^] calcd for Chemical Formula: C_32_H_29_NS, 459.20, found, 459.24. ^1^H NMR (600 MHz, Chloroform-*d*) *δ* 8.22 – 8.17 (m, 1H), 8.10 (s, 1H), 7.96 (s, 1H), 7.81 - 7.75 (m, 1H), 7.71 - 7.67 (m, 2H), 7.67 - 7.62 (m, 2H), 7.62 - 7.58 (m, 1H), 7.43 (dtd, *J* = 20.7, 7.3, 1.2 Hz, 2H), 7.35 (s, 1H), 7.13 (s, 1H), 1.74 - 1.64 (m, 4H), 1.35 (s, 6H), 1.08 (s, 6H).

Figure S15. Mass spectrum of **Th-Cz-BN6-C**.

Figure S16. ^1^H NMR spectrum of **Th-Cz-BN6-C**.

Synthesis of 13-(4-chloro-5-(3,6-di-*tert*-butyl-9*H*-carbazol-9-yl)-[1,1'-biphenyl]-3-yl)-8,8,11,11-tetramethyl-6-phenyl-9,10,11,13-tetrahydro-8*H*-benzo[*b*]benzo[4,5]thieno[2,3-*h*]carbazole (Th-Cz-BN6-D): Th-Cz-BN6-C (2.7 g, 5.88 mmol), 3,6-di-*tert*-butyl-9-(4-chloro-5-fluoro-[1,1'-biphenyl]-3-yl)-9*H*-carbazole (2.84 g, 5.88 mmol), and Cs₂CO₃ (2.87 g, 8.82 mmol) were dissolved in 20 mL of DMF. The mixture was heated to 150°C under a nitrogen atmosphere with stirring for 8 hours. After completion of the reaction and cooling to room temperature, the mixture was poured into an aqueous ammonium chloride solution, filtered, and the filter cake was collected and purified by column chromatography. The target molecule was obtained as a white solid of 3.03 g with a yield of 56.0%. EI-MS (m/z): [M^+^] calcd for C_64_H_59_ClN_2_S, 922.41, found, 922.56. ^1^H NMR (600 MHz, Chloroform-*d*) *δ* 8.24 - 8.18 (m, 3H), 8.05 (d, *J* = 2.2 Hz, 1H), 8.03 (d, *J* = 2.2 Hz, 1H), 8.00 (s, 1H), 7.81 - 7.77 (m, 1H), 7.75 (ddt, *J* = 6.4, 3.2, 1.9 Hz, 2H), 7.71 - 7.58 (m, 6H), 7.52 (dd, *J* = 8.6, 1.9 Hz, 1H), 7.49 - 7.38 (m, 6H), 7.31 (d, *J* = 8.5 Hz, 1H), 7.21 (d, *J* = 8.4 Hz, 2H), 1.77 - 1.66 (m, 4H), 1.49 (d, *J* = 8.3 Hz, 18H), 1.37 (d, *J* = 12.8 Hz, 6H).

Figure S17. Mass spectrum of **Th-Cz-BN6-D**.

Figure S18 ^1^H NMR spectrum of **Th-Cz-BN6-D**.

**Synthesis of Th-Cz-BN6: Th-Cz-BN6-D** (3.00 g，3.25 mmol) was dissolved in 15.0 mL of *tert*-butylbenzene. Under nitrogen atmosphere, *t*-BuLi (1.3 M, 5.0 mL, 6.5 mmol) was added dropwise at -78 ℃ and stirred for 0.5 h, the temperature was raised to 70°C, and stirred for 2 hours. The reaction was then cooled to -40 ℃, and BBr_3_ (1.63 g, 6.5 mmol) was added dropwise. The mixture was allowed to warm to room temperature and stirred for 1 hour. Subsequently, *N*,*N*-diisopropylethylamine ( 0.84 g, 6.5 mmol) was added dropwise at 0 ℃, and the reaction system was heated to 150 ℃ with stirring for 6 hours. Upon completion of the reaction, the mixture was quenched by adding 2.0 mL each of methanol and water. The reaction mixture was extracted with dichloromethane and water. The organic phase was concentrated under reduced pressure and purified by column chromatography, The target molecule was obtained as a yellow solid of 1.31 g with a yield of 45.0%. EI-MS (m/z): [M^+^] calcd for C_64_H_57_BN_2_S, 896.43, found, 896.49. ^1^H NMR (600 MHz, Chloroform-*d*) *δ* 8.59 (s, 1H), 8.53 - 8.44 (m, 3H), 8.38 (d, *J* = 1.8 Hz, 1H), 8.30 (d, *J* = 2.0 Hz, 1H), 8.29 - 8.23 (m, 2H), 8.07 - 8.01 (m, 2H), 7.87 - 7.79 (m, 3H), 7.77 - 7.63 (m, 6H), 7.56 - 7.52 (m, 1H), 7.32 (ddd, *J* = 8.0, 7.0, 1.2 Hz, 1H), 7.24 (s, 1H), 6.97 (ddd, *J* = 8.2, 6.9, 1.1 Hz, 1H), 1.86 - 1.69 (m, 4H), 1.58 (s, 3H), 1.56 (s, 9H), 1.37 (s, 3H), 1.20 (s, 9H), 1.14 (s, 3H), 1.12 (s, 3H). ^13^C NMR (151 MHz, Chloroform-*d*) *δ* 145.50, 145.37, 144.68, 143.86, 143.38, 143.23, 142.16, 141.73, 140.87, 139.59, 139.40, 138.64, 138.61, 138.42, 137.56, 136.20, 134.67, 131.73, 129.54, 129.25, 129.23, 129.16, 129.13, 128.90, 128.50, 128.30, 127.75, 127.55, 126.04, 124.43, 123.83, 123.61, 123.10, 122.49, 121.40, 121.04, 120.90, 117.43, 114.40, 110.80, 107.38, 106.17, 35.23, 35.01, 34.86, 34.33, 32.80, 32.38, 32.24, 31.87, 31.83, 31.56.

Figure S19. Mass spectrum of **Th-Cz-BN6**.

Figure S20. ^1^H NMR spectrum of **Th-Cz-BN6**.

Figure S21. ^13^C NMR spectrum of **Th-Cz-BN6**.

Figure S22. Absolute configurations of Th-Cz-BN6 in crystals.

Figure S23. TGA and DSC curves of Th-Cz-BN3 and Th-Cz-BN6.

Figure S24. CV curves of Th-Cz-BN3 and Th-Cz-BN6.

Figure S25. The hole (blue isosurface) and electron (green isosurface) distribution of different excited states.

Figure S26. The hole and electron distribution and overlap on different fragments of ThCz-BN3.

Figure S27. The hole and electron distribution and overlap on different fragments of ThCz-BN6.

Figure S28. Reorganization energies of Th-Cz-BN3 (a) and Th-Cz-BN6 (b).

Figure S29. Reorganization energies of Th-Cz-BN3 and Th-Cz-BN6 at different frequencies (a). (b) The main vibrational mode and reorganization energies of Th-Cz-BN3. (c) The main vibrational mode and reorganization energies of Th-Cz-BN6.

Figure S30. Huang-Rhys factors of Th-Cz-BN3 and Th-Cz-BN6 at different frequencies (a). (b) The main vibrational mode and reorganization energies of Th-Cz-BN3. (c) The main vibrational mode and reorganization energies of Th-Cz-BN6.

Figure S31. PL and phosphorescence spectra in solutions at 77 K of Th-Cz-BN3 (a) and Th-Cz-BN6 (b).

Figure S32. PL spectra of ThCz-BN3 (a) and ThCz-BN6 (b) in solutions with different ratios of tetrahydrofuran and water. (3) PL spectra of ThCz-BN6 in THF with different concentrations. Experimental method: the emitters were dissolved in THF with a concentration of 10^-4^ M. A 100% THF sample was composed of 0.3 mL solutions (emitters in THF) and 2.7 mL pure THF. An 80% THF sample was composed of 0.3 mL solutions (emitters in THF), 2.1 mL pure THF, and 0.6 mL water. A 60% THF sample was composed of 0.3 mL solutions (emitters in THF), 1.5 mL pure THF, and 1.2 mL water. A 40% THF sample was composed of 0.3 mL solutions (emitters in THF), 0.9 mL pure THF, and 1.8 mL water. A 20% THF sample was composed of 0.3 mL solutions (emitters in THF), 0.3 mL pure THF, and 2.4 mL water. A 10% THF sample was composed of 0.3 mL solutions (emitters in THF) and 2.7 mL water.

Figure S33. (a) PL spectra of PhCzBCz and absorption spectra of Th-Cz-BN3 and Th-Cz-BN6. (b) Transient PL spectrum of PhCzBCz.

Figure S34. Temperature-dependent transient decay curves of Th-Cz-BN3 and Th-Cz-BN6-based films.

Figure S35. EL spectra (Inset: CIE coordinates of the EL spectra) (a), luminance-current density-voltage curves (b), EQE-luminance (c), PE-CE-luminance (d) of the Th-Cz-BN3-based devices with different doping concentrations.

Figure S36. EL spectra (Inset: CIE coordinates of the EL spectra) (a), luminance-current density-voltage curves (b), EQE-luminance (c), PE-CE-luminance (d) of the Th-Cz-BN6-based devices with different doping concentrations.

Figure S37. The refractive index (n) and extinction coefficient (k) at different wavelengths of the films.

Figure S38. *p*-Polarized angle-dependent PL radiance of the PhCzBCz: 3.0 wt% Th-Cz-BN3 (a) and Th-Cz-BN6 (b) films.

Figure S39. The angle-dependent EL spectra of the devices (host: PhCzBCz).

Figure S40. Transient EL spectra of (a) Th-Cz-BN3 and (b) Th-Cz-BN6-based devices (host: PhCzBCz) under 10 mA cm^-2^.

Figure S41. Device structure of the phosphor-sensitized devices, as well as the chemical structures used in device fabrication. The device structures were [ITO/ BPBPA (50 nm)/ Prime (10 nm)/ GH: 5.0 wt% GD: 1.0 wt% ThCz-BN3 or ThCz-BN6 (40 nm)/ TRZ-DBF (5 nm)/ NA-An-Im (35 nm)/ Liq (2 nm)/ Al (100 nm)]

Figure S42. EL properties of the devices (host: GH).

Figure S43. The operational lifetime of the devices with GH as the host.

Figure S44. Transient EL spectra of Th-Cz-BN3 and Th-Cz-BN6 with GH as the host.

Figure S45. EL properties of the phosphor-sensitized devices.

Figure S46. The refractive index (n) and extinction coefficient (k) at different wavelengths of the films.

Figure S47. *p*-Polarized angle-dependent PL radiance of the sensitized films of Th-Cz-BN3 (a) and Th-Cz-BN6 (b).

Figure S48. Lifetimes of Th-Cz-BN3 and Th-Cz-BN6-based PSF devices.

Figure S49. Transient EL spectra of (a) Th-Cz-BN3 and (b) Th-Cz-BN6-based PSF devices under 10 mA cm^-2^.

Figure S50. Time-resolved emission spectra of Th-Cz-BN3 and Th-Cz-BN6 in the sensitized films. The initial emission intensity of the two films was the same.

Figure S51. CV curves of Th-Cz-BN3 and Th-Cz-6 (20 cycles).


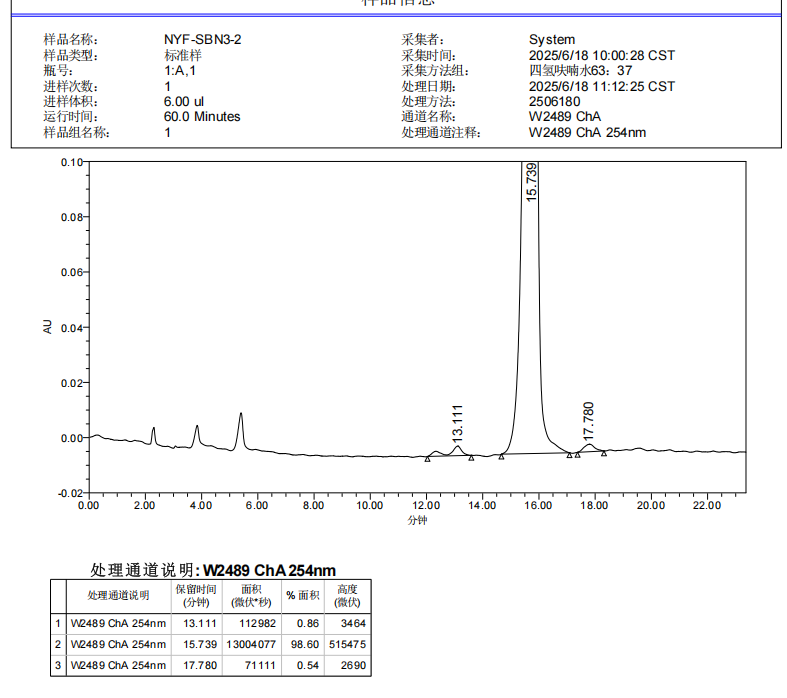


Figure S52. HPLC spectra of Th-Cz-BN3.


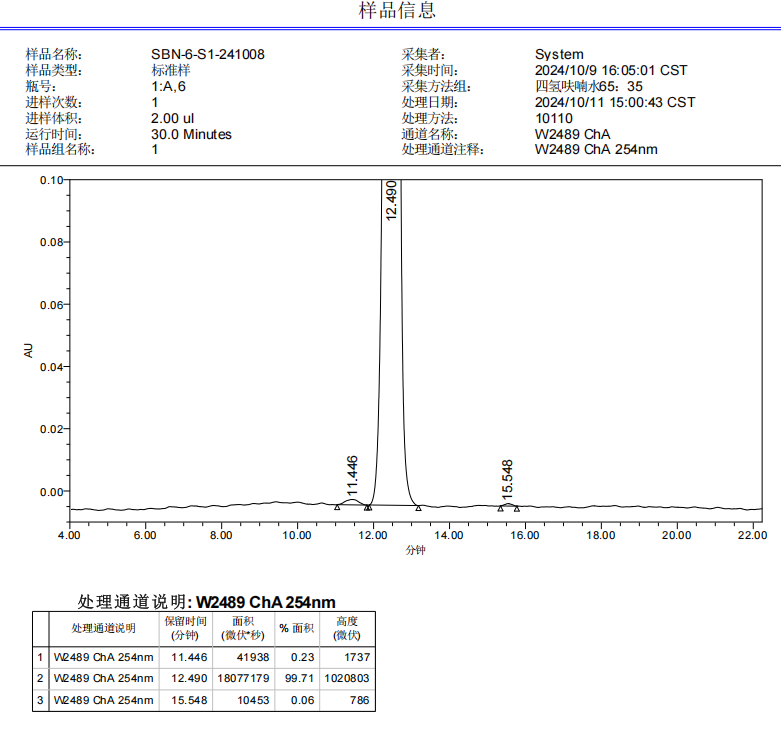


Figure S53. HPLC spectra of Th-Cz-BN6.

Table S1. The contributions of sulphur atoms in transitions of the ground and excited states.

| Compounds | S_0_ | | T_1_ | | T_2_ | | S_1_ | |
| --- | --- | --- | --- | --- | --- | --- | --- | --- |
|  | HOMO | LUMO | hole | particle | hole | particle | hole | particle |
| ThCz-BN3 | 4.74% | 0.74% | 4.37% | 0.75% | 8.83% | 1.15% | 4.51% | 0.71% |
| ThCz-BN6 | 6.76% | 1.06% | 6.73% | 0.94% | 2.33% | 1.77% | 5.92% | 1.04% |

Table S2. Hole and electron analysis of the excited states of Th-Cz-BN3.

| excited states | energy levels (eV) | Sr index (a.u.) | D-index (Å) | Δσ index (Å) | H_CT (Å) | H index (Å) | t index (Å) | SOC constants with S_1_ (cm^-1^) |
| --- | --- | --- | --- | --- | --- | --- | --- | --- |
| T_1_ | 2.222 | 0.6516 | 0.6 | 0.044 | 2.650 | 4.278 | -2.049 | 0.05 |
| T_2_ | 2.386 | 0.7766 | 0.953 | 0.401 | 2.692 | 3.762 | -1.739 | 0.49 |
| T_3_ | 2.767 | 0.7729 | 1.248 | 0.153 | 3.423 | 4.294 | -2.175 | 0.26 |
| T_4_ | 2.836 | 0.8591 | 0.897 | -0.103 | 3.926 | 4.807 | -3.028 | 0.15 |
| T_5_ | 2.938 | 0.8555 | 0.543 | 0.125 | 3.049 | 4.412 | -2.506 | 0.28 |
| S_1_ | 2.597 | 0.6217 | 1.018 | 0.114 | 2.604 | 4.301 | -1.586 | - |

Table S3. Hole and electron analysis of the excited states of Th-Cz-BN6.

| excited states | energy levels (eV) | Sr index (a.u.) | D-index (Å) | Δσ index (Å) | H_CT (Å) | H index (Å) | t index (Å) | SOC constants with S_1_ (cm^-1^) |
| --- | --- | --- | --- | --- | --- | --- | --- | --- |
| T_1_ | 2.174 | 0.6623 | 0.523 | -0.170 | 2.604 | 4.101 | -2.081 | 0.14 |
| T_2_ | 2.369 | 0.7920 | 0.769 | 0.150 | 2.550 | 3.847 | -1.781 | 0.35 |
| T_3_ | 2.719 | 0.8142 | 1.350 | 0.451 | 2.848 | 3.811 | -1.498 | 0.26 |
| T_4_ | 2.765 | 0.7423 | 0.824 | -0.155 | 3.157 | 4.273 | -2.333 | 0.27 |
| T_5_ | 2.935 | 0.8295 | 0.448 | -0.103 | 3.087 | 4.311 | -2.638 | 0.30 |
| S_1_ | 2.549 | 0.6241 | 1.062 | -0.072 | 2.677 | 4.181 | -1.615 | - |

Table S4. The relevant parameters in the calculation of *k*_FRET_.

| Emitter | J  (×10^14^ M^-1^cm^-1^nm^4^) | *k*^2^ | φ_PLQY,D_ | n | R_0_  (Å) | E | r  (Å) | *k*_FRET_  (×10^7^ s^-1^) |
| --- | --- | --- | --- | --- | --- | --- | --- | --- |
| Th-Cz-BN3 | 2.525 | 0.843 | 0.24 | 1.769 | 27.8 | 18.4% | 35.6 | 6.0 |
| Th-Cz-BN6 | 2.989 | 0.846 | 0.24 | 1.750 | 28.8 | 23.7% | 35.1 | 8.0 |

Table S5. A comparison of MR-TADF emitter-based non-sensitized devices with CIEy over 0.7.

| Emitter | *λ*_EL_ (nm) | FWHM (nm) | CIE(x,y) | EQE_max_ (%) | EQE_1000_ (%) | Roll-off (%) |
| --- | --- | --- | --- | --- | --- | --- |
| Th-Cz-BN3 | 525 | 30 | (0.24, 0.71) | 36.4 | 30.3 | 18.3 |
| Th-Cz-BN6 | 529 | 29 | (0.24, 0.72) | 34.2 | 15.5 | 57.7 |
| DBTN-2 | 520 | 29 | (0.19, 0.74) | 35.2 | 20.4 | 42.0 |
| NT-2BN | 517 | 21.5 | (0.17, 0.74) | 30.5 | 17.8 | 41.6 |
| BN-STO | 517 | 29 | (0.19, 0.70) | 40.1 | 28.1 | 29.9 |
| BN-XTO | 516 | 29 | (0.19, 0.70) | 37.3 | 18.6 | 50.1 |
| TRZTPh-BNCz | 513 | 33 | (0.16, 0.70) | 31.4 | 23.1 | 26.4 |
| D-TCz-VTCzBN | 524 | 37 | (0.22, 0.71) | 32.2 | 16 | 50.3 |
| BN-TP | 528 | 36 | (0.26, 0.70) | 35.1 | 20.8 | 40.7 |
| *m*-CzB | 515 | 39 | (0.20, 0.70) | 23.5 | 15 | 36.2 |
| TCZBAO | 520 | 36 | (0.20, 0.70) | 25.1 | 14 | 44.2 |
| BN-ICz-1 | 523 | 23 | (0.22, 0.74) | 30.5 | 12.8 | 58.0 |
| BN-ICz-2 | 523 | 23 | (0.23, 0.73) | 29.8 | 22 | 26.2 |
| ω-DABNA | 512 | 25 | (0.13, 0.73) | 30.1 | 29.4 | 2.3 |
| ω-DABNA-M | 515 | 25 | (0.15, 0.74) | 32.7 | 30.3 | 2.4 |
| ω-DABNA-Ph | 521 | 30 | (0.19, 0.74) | 32.7 | 27.4 | 16.2 |

Table S6. The summary properties of the exciplex co-host-based devices.

| Coumpound | V_on_  [V] | λ_em_  [nm] | FWHM  [nm] | CE_max_  [cd A^-1^] | PE_max_  [lm W^-1^] | EQE^[a]^  [%] | CIE(x,y) | Lifetime  @1000 cd m^-2^ (h) |
| --- | --- | --- | --- | --- | --- | --- | --- | --- |
| Th-Cz-BN3 | 2.4 | 529 | 31 | 104.3 | 131.9 | 24.9, 23.1, 15.7 | (0.24, 0.72) | 50.0 |
| ThCz-BN6 | 2.4 | 533 | 30 | 103.1 | 134.5 | 24.0, 17.0, 6.1 | (0.27, 0.70) | 239.1 |
| ThCz-BN3  (Sensitized) | 2.2 | 529 | 31 | 112.0 | 159.9 | 27.4, 25.8, 22.0 | (0.26, 0.70) | 1083.7  (LT_95_) |
| ThCz-BN6  (Sensitized) | 2.2 | 533 | 30 | 115.9 | 165.5 | 27.4, 23.5, 16.3 | (0.28, 0.69) | 5895.2  (LT_95_) |

[a] The maximum EQE and the values at 1000 and 10000 cd m^-2^.

Reference

[S1] R. Krishnan, J. S. Binkley, R. Seeger, J. A. Pople, "Self‐consistent molecular orbital methods. XX. A basis set for correlated wave functions" *J Chem Phys* **1980**, *72*, 650-654.

[S2] A. D. Becke, "Density‐functional thermochemistry. III. The role of exact exchange" *J Chem Phys* **1993**, *98*, 5648-5652.

[S3] Gaussian 09 Rev. E.01, M. J. Frisch, G. W. Trucks, H. B. Schlegel, G. E. Scuseria, M. A.Robb, J. R. Cheeseman, G. Scalmani, V. Barone, G. A. Petersson, H. Nakatsuji, X. Li, M. Caricato, A. V. Marenich,J. Bloino, B. G. Janesko, R. Gomperts, B. Mennucci, H. P. Hratchian, J. V. Ortiz, A. F. Izmaylov, J. L. Sonnenberg, Williams, F. Ding, F. Lipparini, F. Egidi, J. Goings, B. Peng, A. Petrone, T. Henderson, D. Ranasinghe, V. G.Zakrzewski, J. Gao, N. Rega, G. Zheng, W. Liang, M. Hada, M. Ehara, K. Toyota, R. Fukuda, J. Hasegawa, M.Ishida, T. Nakajima, Y. Honda, O. Kitao, H. Nakai, T. Vreven, K. Throssell, J. A. Montgomery Jr., J. E. Peralta, F.Ogliaro, M. J. Bearpark, J. J. Heyd, E. N. Brothers, K. N. Kudin, V. N. Staroverov, T. A. Keith, R. Kobayashi, J.Normand, K. Raghavachari, A. P. Rendell, J. C. Burant, S. S. Iyengar, J. Tomasi, M. Cossi, J. M. Millam, M. Klene, C. Adamo, R. Cammi, J. W. Ochterski, R. L. Martin, K. Morokuma, O. Farkas, J. B. Foresman, D. J. Fox, Gaussian, Inc., Wallingford CT, **2009**.

[S4] T. Lu, F. Chen, "Multiwfn: A multifunctional wavefunction analyzer" *J. Comput. Chem.* **2012**, *33*, 580-592.

[S5] F. Neese, "Software update: the ORCA program system, version 4.0" *WIREs Comput. Mol. Sci.* **2018**, *8*, e1327.

[S6] Y. X. Hu, J. Miao, T. Hua, Z. Huang, Y. Qi, Y. Zou, Y. Qiu, H. Xia, H. Liu, X. Cao, C. Yang, "Efficient selenium-integrated TADF OLEDs with reduced roll-off" *Nat. Photonics* **2022**, *16*, 803-810.
